# A Critical Review on Phytochemical Profile and Biological Effects of Turnip (*Brassica rapa L*.)

**DOI:** 10.3389/fnut.2021.721733

**Published:** 2021-07-29

**Authors:** Qingsui Cao, Ge Wang, Ye Peng

**Affiliations:** ^1^Institute of Agricultural Facilities and Equipment, Jiangsu Academy of Agricultural Sciences, Nanjing, China; ^2^School of Food and Biological Engineering, Jiangsu University, Zhenjiang, China

**Keywords:** turnip, *Brassica rapa L*, bioactivities, chemical compositions, molecular mechanisms

## Abstract

A growing number of medicinal and edible plants have attracted increasing attention due to their abundant constituents and biological activities including turnip. Turnip (*Brassica rapa L*.) is an herbaceous biennial plant belonging to Cruciferae Brassica. As one of the oldest cultivated vegetables widely consumed in Asia, the turnip has received significant attention in the studies of its bioactive components and biological function. Multiple bioactive components in turnip, such as glucosinolates, isothiocyanate, phenolic compounds, flavonoids, and organic acids, were identified. The bioactivity studies on turnip revealed its anticancer, antimicrobe, anti-hypoxia, anti-diabetes, anti-oxidation, and nephroprotective activity. The present review mainly summarized the previous studies on the chemical compositions of turnip and the bioactivities associated with turnip. Further studies on the extraction and purification of compounds from a turnip as well as its potential molecular mechanisms are highly needed to utilize turnip as a functional food plant in a better way.

## Introduction

Recently, an increasing number of medicinal and/or edible plants have received more attention due to their multiple bioactive effects. Cruciferous vegetables, in particular the Brassica genus, including broccoli, cabbage, and turnip, are widely consumed for medicinal treatments in the past few decades. The vegetables of the Brassica genus are an adequate source of glucosinolates, which can be hydrolyzed into isothiocyanates *via* the plant enzyme myrosinase (1).

Turnip, also named *Brassica rapa L*. in Latin, is defined as a biennial herbaceous plant belonging to Cruciferae Brassica which matures in 2 months and can be planted in the spring, late summer, and fall for roots or greens (1, 2). Turnip is one of the oldest cultivated vegetables and has been widely consumed in high-altitude localities in Asian countries (3). For example, the root of turnip is eaten by Tibetan nationality people living in Qinghai-Tibet Plateau for fatigue relief and hypoxia prevention (4). One research on its chemical composition revealed that this plant is a rich resource of glucosinolates (1, 5, 6), phenolic compounds, organic acids (7, 8), flavonoids (9), sulfur compounds (10), and volatiles (2, 11, 12), which has been found to contribute to different health-beneficial effects. A few studies revealed that turnip exhibits the bioactivities including anti-cancer (1, 2, 13), anti-bacteria (1, 7), anti-oxidation (6–8, 14), anti-radiation (15), and anti-diabetes (16, 17). More recently, several novel biological effects, such as nephroprotection (18), hypoxia cerebral and pulmonary edema prevention (19, 20), and osteoporosis prevention (15), have been reported to enrich turnip as a functional food.

To better understand the beneficial effects of turnip as a natural health tonic, literature associated with turnip were reviewed according to the Web of Science Core Collection. This current review first introduced the chemical composition of turnip, followed by the discussion of the biological activities of turnip and the factors affecting the bioactive components of turnip.

## Chemical Composition of Turnip

### Nutrient Substances

The chemical composition analysis of the root of turnip showed that it is rich in multiple nutritional profiles including carbohydrate, protein, dietary fiber, vitamin C, essential amino acid, and mineral element, but less fat (21). Turnip leaves possess more nutrients compared with turnip roots. Besides the nutrients mentioned above, some researchers reported that turnip leaves are abundant sources of carotenoids, lutein, vitamin A, vitamin B, and vitamin K (22).

### Glucosinolates and Isothiocyanate

Glucosinolates are highly present in turnip with three subunits including glucose, amino acid, and sulfate. Glucosinolates have been classified into aliphatic glucosinolates, aromatic glucosinolates, and indole glucosinolates according to the different amino acid constituents (21). They can be rapidly hydrolyzed by myrosinase (i.e., β-thioglucosidase, thioglucoside glucoside glucohydrolase, EC 3.2.3.1) into various metabolites including glucose, sulfate, isothiocyanate, nitrile, and thiocyanate, which have been found to exhibit multiple biological activities (1, 2, 5). A total of 10 glucosinolates were determined by the comparative analysis of 59 varieties of turnip tubers from 4 agroclimatic zones in China. Among them, gluconasturtiin and glucobrassicanapin are the two main glucosinolates with the highest contents (21). Compared with turnip roots, turnip leaves contain more types of glucosinolates. The study from Padilla et al. (23) showed that turnip greens contain 16 kinds of glucosinolates in a collection of 113 varieties of turnip from northwestern Spain. Among them, the levels of aliphatic glucosinolates, such as gluconapin and glucobrassicanapin, are the most abundant, but indolic glucosinolate and aromatic glucosinolate concentrations are relatively low (24). Besides, the sensory analysis on the flavor of turnip indicated that the glucosinolate concentration is closely associated with the taste of bitterness (2, 12, 23). Turnip has also been reported to be a natural source of isothiocyanates including sulforaphane (10). The total content of glucosinolates in different parts of the turnip (peeled root, peeling, and leaf) ranges from 147 to 151 μmol/100 g, suggesting no significant difference among these parts. In addition, 3-butenly, 4-pentenyl, and β-phenylethyl isothiocyanate are the three isothiocyanates with high contents based on GC/MS and GC analyses (1, 11, 12).

### Phenolic Compounds and Organic Acids

Phenolic compounds and organic acids, two known bioactive compounds wildly present in plants, have been reported to possess many health benefits, including antioxidation, anti-inflammation, anti-diabetes, and neuroprotection. Many studies have detailly reported the 14 phenolic compounds and 6 organic acids in turnip (7, 8). The phenolic compounds include 3-p-coumaroylquinic acid, caffeic acid, ferulic acid, sinapic acid, kaempferol 3-O-sophoroside-7-O-glucoside, kaempferol 3-O-sophoroside-7-O-sophoroside, kaempferol 3-O-(feruloyl/caffeoyl)-sophoroside-7-O-glucoside, kaempferol 3,7-O-diglucoside, isorhamnetin 3,7-O-diglucoside, kaempferol 3-O-sophoroside, 1,2-disinapoylgentiobiose, 1,20 -disinapoyl-2-feruloylgentiobiose, kaempferol 3-O-glucoside, and isorhamnetin 3-O-glucoside. Among them, the quantification analysis revealed that kaempferol 3-O-sophoroside-7-O-glucoside, kaempferol 3-O-(feruloyl/caffeoyl)-sophoroside-7-O-glucoside, isorhamnetin 3,7-O-diglucoside, and isorhamnetin 3-O-glucoside are the main phenolics. The contents of phenolic compounds in turnip exhibited a high level (about 18 g/kg), more than two times as much as those in *Brassica oleracea L*. var. *acephala* (7, 8). El-Makawy and his colleagues (15) reported that the total phenolic content in turnip oil was detected at 4.1 mg GAE/g. Another study conducted by Romani et al. (9) revealed that turnip top contains a high level of polyphenol compounds, especially flavonoids (about 119.2–138.85 mg/100 g), which is about 3–10 times than other Brassica genus. The contents of hydroxycinnamic derivatives were found at the level of 5.77–52.54 mg/100 g. It is also reported that all flavonoids in turnip top are glycosylated derivatives of isorhamnetin, kaempferol, and quercetin glycosides (9). In addition, three new chalcone glycosides, that is, 4′-O-β-D-glucopyranosyl-4-hydroxy-3′-methoxychalcone, 4′-O-β-D-glucopyranosyl-3′, 4-dimethoxychalcone, and 4,4′ -di-O-β-D-glucopyranosyl-3′-methoxychalcone, have been isolated from the aerial parts of turnip (25). The organic acids in turnip include malic acid, aconitic acid, citric acid, ketoglutaric acid, shikimic acid, and fumaric acid mostly found in turnip leaves, stems, and flower buds. Among them, malic acid is the dominant one (7, 8). The total organic acid content in the flower buds and leaves and stems of turnip showed a high level (ranging from 36 to 51 g/kg, dry basis), which was significantly higher than that of roots. Although malic acid is the dominant one in three turnip edible parts, the ratio of it is significantly different: the roots showed the highest level of malic acid (81%), followed by leaves and stems (65%), and flower buds exhibited a lower level (41%) (8). Shikimic acid is the least one, accounting for only 0.1–0.3% of the total acid. In addition, the content of aconitic acid in flower buds was the highest (14%), which was significantly higher than that in roots (2%) (8). It should be pointed that ferulic acid and synaptic acid are only detected in turnip roots (8).

### Sugar

The total concentration of soluble sugar in turnip (including leaf and bulb) is about 345 g/kg, which is lower than that of swede but higher than that of kale and rape. The primary soluble sugars are glucose, fructose, and sucrose (26). The results are consistent with a previous study conducted by Hong and Kim (1).

### Volatiles

A series of volatiles exist in Brassica plants, including glucosinolates, alcohol, esters, aldehydes, terpenes, and ketones. These components are supposed to defend against herbivore attacks. In addition, plant volatiles are also considered to have great beneficial effects on human health (12, 27). The composition of volatiles in turnip at different developmental stages (seeds, sprouts with 6 and 9 days of age, and adult plants) was detected in 2009 (12). It showed that a total of 64 volatile compounds were characterized, including 1 alcohol, 15 aldehydes, 10 esters, 1 ketone, 1 nitrogen compound, 12 norisoprenoids, 14 sulphury compounds, 8 terpenes, and 2 miscellaneous compounds. Among them, 47 components were found in the adult plant, 37 and 41 components in sprouts with 6 and 9 days, respectively, and 18 components in seeds. Isothiocyanates were the main volatiles in all materials, in which 3-butenyl isothiocyanate was the dominant one. Terpenes, aldehydes, norisoprenoids, and ester compounds were exhibited at higher levels in sprouts within 9 days of development (12); however, Xue et al. (11) detected a total of 67 volatiles in fresh turnips and dried turnip chips. Esters are the predominant volatiles (92.51%), especially isothiocyanato-cyclopropane (37.30%) and (2-isothiocyanatoethyl)-benzene (28.35%). On the other hand, there were around 15–20 kinds of volatiles detected in turnip chips dried by freeze-drying, explosion puff drying, infrared drying, and hot air-drying, respectively. Interestingly, besides 2-methyl-propanoic acid and 1-(1,1-dimethylethyl)-2-methyl-1,3-propanediyl ester, other volatile components in turnip chips with different drying methods are observed, which are significantly different from each other. This finding suggests that different drying methods result in the generation of different volatiles, which should be considered as a major factor for the flavor and quality of turnip chips.

## Biological Activities of Turnip

### Anti-cancer Activity

Cancer is one of the most serious chronic diseases threatening human health all over the world, which causes thousands of deaths per year. Many cruciferous vegetables have been found to possess anticancer activity due to their bioactive components including isothiocyanate (13). Isothiocyanate is the breakdown product of glucosinolates. Due to the high content of glucosinolates in turnip, this plant has been found to reduce the risk of cancer effectively (1, 28). β-phenylethyl isothiocyanate is the most dominant isothiocyanate in turnip (11). One study showed that the growth of human-derived hepatoma cells (HepG2) was significantly inhibited with the treatment of β-phenylethyl isothiocyanate in a concentration-dependent manner (1). Another study indicated that the treatment of β-phenylethyl isothiocyanate significantly inhibited the growth of the human prostate cancer cell line DU145 *via* the inhibition of the JAK/STAT3 pathway (29).

### Antimicrobial Activity

Bacterial infection is a major risk factor for human health since it can induce diarrhea, vomit, enteritis, and even death. Antibiotics are widely used as specific drugs for the treatment of bacterial infections; however, drug resistance and side effects of antibiotics are considered a big concern. Thus, natural alternatives for the treatment of pathogenic bacterial infections are imperiously needed. Turnip has been reported to be a source of safe antimicrobial compounds (30, 31). Methanol extracts of turnip including carbon tetrachloride and chloroform soluble fractions have been found to inhibit nine genus bacteria including three Gram-positive bacteria (*Bacillus cereus, B. subtilis, and Sarcina lutea*) and 6 Gram-negative bacteria (*Escherichia coli, Pseudomonas aeruginosa, Salmonella Paratyphi, S. Typhi, S. dysenteriae, and Vibrio parahemolyticus*), suggesting an effective antimicrobial activity of turnip (30). Another animal study reported the inhibitory effect of turnip root on *Helicobacter pylori*. Mice treated with a high dose of myrosinase-reacted turnip roots showed a significant anti-*H. pylori* effect, which was even comparable to that of amoxicillin. Besides, the serum of mice fed with a high dose of turnip roots lyophilized powder (200 mg/Kg/day) exhibited a higher level of anti-*H. pylori* antibody. The active components are considered to be isothiocyanates, and the metabolites of glucosinolates are decomposed by myrosinase in turnip. This study indicates that turnip may act as the potential functional food for the treatment of *H. pylori* infection (32). Another study also reported that β-phenylethyl isothiocyanate found in turnip root significantly attenuated the viability of *V. parahaemolyticus, Staphylococcus aureus*, as well as *B. cereus* (1). Interestingly, the inhibitory effects of β-phenylethyl isothiocyanate on these bacteria might be different. Sousa and his colleagues (7) found that the aqueous extracts of turnip inflorescences, including β-phenylethyl isothiocyanate, exhibit different levels of inhibitory effect on different bacteria, with the order of *S. aureus* > *B. cereus* >> *B. subtilis*. An *in vitro* experiment conducted by Aires et al. (33) suggested that β-phenylethyl isothiocyanate intensely inhibited the activity of Gram-positive bacteria, including *S. aureus* and *S. saprophyticus*, which were isolated from human and pig gastrointestinal segments. β-phenylethyl isothiocyanate showed a relatively low inhibitory activity on *Enterobacter hormaechei, E. coli strains, Klebsiella oxytoca, Morganella morganii, P. aeruginosa*, and S*tenotrophomonas maltophilia*. All the above findings indicate that turnip can be considered a preventive tool for bacterial infection.

### Anti-hypoxia Activity

Oxygen is essential for humans to sustain life. Exposure to a low oxygen environment, such as high-altitude localities, may induce a series of stress responses including breath shortness, increased blood pressure, and faster heart rate. Thus, finding some naturally occurring food that possesses a beneficial effect on hypoxia is important. Turnip root has been historically used for hypoxia relief for a long time by Tibetan nationality people, who lived in Qinghai-Tibet Plateau (4, 34). One human study conducted by Chu et al. revealed that human hypoxia-tolerance was significantly improved with a 7-day Tibetan turnip consumption, through promoting oxygen absorption in red blood cells and oxygen transportation with hemoglobin (34). Several studies suggested that polysaccharides, p-coumaric acid, and p-coumaric acid-β-D-glucopyranoside in turnip are potentially active components contributing to the anti-hypoxia effect of turnip (4, 19, 20). Within these compounds, p-coumaric acid and p-coumaric acid-β-D-glucopyranoside have been found to protect mice from hypoxia pulmonary edema and hypoxia cerebral edema. As these studies used male Kunming mice as an animal model, further human studies are required to determine effective compounds in turnip on promoting hypoxia tolerance (4).

### Antidiabetic Activity

Diabetes is a chronic and metabolic disorder typically characterized by high levels of blood glucose and impaired glucose metabolism, which induces an increased public concern worldwide (35). Although some drugs, including insulin, metformin, and glucosidase inhibitors, have been widely used for diabetic treatments, their negative effects, such as drug resistance, side effects, and even painful experience, are big concerns that drive researchers to find safer alternatives for the prevention and/or treatment of diabetes (2). Jung et al. (16) reported that ethanol extracts of turnip root improved glucose transportation and ameliorated insulin resistance *via* reducing the levels of blood glycosylated hemoglobin, plasma insulin, C-peptide, as well as glucagon in type 2 diabetic db/db mice. They also found that turnip extracts promoted the insulin/glucagon ratio and hepatic glycogen content by the promotion of hepatic glucose regulating enzyme activity (16). Furthermore, both ethyl acetate extracts and butanol extracts of turnip were found to effectively inhibit α-glucosidase activity. Twelve compounds were isolated and identified from ethyl acetate extracts and butanol extracts, two of them, namely licochalcone A and caffeic acid, have been revealed to exhibit an inhibitory effect on α-glucosidase.

### Antioxidant and Antiradical Activity

Oxidative stress resulted from excessive reactive oxygen species (ROS) and has been proved to induce many chronic diseases, such as inflammation, neurodegeneration, obesity, and even cancer (36, 37). Many naturally occurring foods and/or their components are considered antioxidants, which can be consumed to defend cellular damage from oxidative stress. Turnip is an abundant source of natural antioxidants, especially phenolic compounds and organic acids (8). The assessment of antioxidant abilities of aqueous extracts of different parts of turnip was obtained by DPPH radical-scavenging assay (8). The result suggested that aqueous extracts of turnip leaves, stems, and flower buds exhibited higher antioxidative capacity, compared with that of turnip roots (8); however, this result is not consistent with another study, which suggested that the aqueous extracts of turnip roots showed higher DPPH radical scavenging activity than that of aqueous extracts of turnip green (31). Different sample collection seasons, different areas, and different subspecies may cause this controversial conclusion. Besides, turnip tops have been reported to possess an effective antiradical capacity probably resulting from a high polyphenols content (about 107–191 mg/100 g in fresh sample) (9). A comparative study among nine cruciferous plants exhibited that turnip sprouts grown for 8 days showed higher antioxidative capacity compared with other varieties. Furthermore, the result showed that there was a positive correlation between the content of phenolic compounds and superoxide radical-scavenging capacity, suggesting that phenolic content can be used as a marker to assess the antioxidation ability of herbals (6). In addition, one study displayed that turnip oil had high DPPH radical scavenging activity. After mixing with DPPH for 1, 30, and 60 min, 10, 19, and 31% of DPPH radicals were scavenged by turnip oil, respectively (15).

### Nephroprotective Activity

Cisplatin (cis-diaminedichloroplatinum II) is considered one of the most efficient chemotherapeutic drugs for the treatment of human solid tumors developing in the head, neck, testis, ovary, and breast; however, the side effect of cisplatin, such as nephrotoxicity, greatly restricts the curative effect of this anticancer drug (2). An investigation of the protective effect of the ethanol extracts of turnip roots against cisplatin-induced nephrotoxicity *in vitro* (LLC-PK1 cells) and *in vivo* (SD male rats) was conducted by Kim and his colleagues in 2006. The result revealed that the intake of the ethanol extracts of turnip roots alleviated cisplatin-induced nephritic injury through the amelioration of oxidative stress (18).

## Factors Affecting the Bioactive Components of Turnip

The investigation of seasonal effects on the composition of bioactive compounds of turnip indicated that the average contents of glucosinolates (glucobrassicanapin, gluconasturtiin, and 4-hydroxyglucobrassicin), total flavonoids, and Vitamin C in turnip roots are higher in the Summer-Winter than those in the Spring-Summer, while less significant changes were found in the total content of phenolics (14).

The elevating atmospheric carbon dioxide is another essential factor that may affect the chemical composition of turnip. Azam et al. (38) found that the increased level of carbon dioxide enhanced the contents of sugar and fiber in turnip; however, the levels of protein, vitamin C, fat, and most amino acids dropped significantly. In addition, several important minerals, such as Ca, Mg, Zn, Mn, and Fe, were also decreased (38). Thus, it is important to maintain the nutritional value of turnip by controlling the release of carbon dioxide into the atmosphere.

To explore the effect of cooking methods on the loss of nutritional components of turnip, Francisco et al. (39) determined the levels of glucosinolates, flavonoids, hydroxycinnamic acids, and vitamin C in turnip cooked by conventional boiling, steaming, and high-pressure cooking. The results showed that conventional boiling and high-pressure cooking induced higher loss of total glucosinolates (more than 60%) and total phenolic content (more than 70%), compared with the loss by steaming method (all below 15%). In addition, the loss of total flavonoids was 5, 64, and 67% by steaming, conventional boiling, and high-pressure cooking, respectively. It indicates that steaming might be a better method to preserve glucosinolates, phenolic compounds, and flavonoids. The level of Vitamin C dropped sharply after cooking, despite the cooking method used (39). Other studies showed that cooking time had a greater influence on the level of ascorbic acid than any cooking method (40), suggesting that the loss of nutrients in turnip could be reduced by controlling cooking time.

## Conclusion

The chemical composition of turnip and its multiple bioactivities, including anticancer, antimicrobial, anti-hypoxia, anti-diabetes, antioxidant, nephroprotective activity, were reviewed in this article. Moreover, the factors affecting bioactive components of turnip were also introduced briefly. To summarize, current studies mainly focus on the identification of various chemical components of turnip and the biological activity of the crude extract of turnip (such as aqueous extract and ethanol extract). As phenolic compounds and sugar are the dominant compounds that exist in turnip, they might be the major factors that contribute to the reported bioactivities of this plant; however, the number of investigations on effective pure substances and underlying molecular mechanisms in turnip is relatively poor. Therefore, the study about the beneficial effects of turnip components might be an interesting area in the future to better understand the relationship between the bioactivity of turnip and chemical constituents. In addition, several *in vitro* (i.e., cell culture) and *in vivo* models (i.e., *Caenorhabditis elegans*, mice, and rats) with the combination of omics techniques are required to study the molecular mechanisms involved, which could systematically explain how turnip and its components exhibit the bioactivities mentioned previously. In total, turnip is considered a beneficial plant with various biological activities and great potential for both medicine and food. Further studies on the detailed bioactivities of turnip are needed to better develop turnip as a functional food.

## Author Contributions

QC and GW performed the draft writing. YP revised the manuscript and proposed the title of the manuscript. All authors contributed to the article and approved the submitted version.

## Conflict of Interest

The authors declare that the research was conducted in the absence of any commercial or financial relationships that could be construed as a potential conflict of interest.

## Publisher's Note

All claims expressed in this article are solely those of the authors and do not necessarily represent those of their affiliated organizations, or those of the publisher, the editors and the reviewers. Any product that may be evaluated in this article, or claim that may be made by its manufacturer, is not guaranteed or endorsed by the publisher.
